# Datasets for a multidimensional analysis connecting clean energy access and social development in sub-Saharan Africa

**DOI:** 10.1016/j.dib.2023.108948

**Published:** 2023-02-07

**Authors:** Paola Casati, Magda Moner-Girona, Ibrahim Khaleel Shehu, Sandor Szabó, Godwell Nhamo

**Affiliations:** a European Commission, Joint Research Centre^1^, Via Enrico Fermi 2749, 21027 Ispra (VA), Italy; b University of Bari, Jonian Department of Law, Economics and Environment, Via Duomo 259, 74100 Taranto, Italy; c African Union Commission, African Union Headquarters, P.O. Box 3243, Roosvelt Street, W21K19,Addis Ababa, Ethiopia; d Institute for Corporate Citizenship, University of South Africa, P.O. Box 392. UNISA 0003, Pretoria, South Africa

**Keywords:** Electricity access, Sustainable development goals, Clean energy, Social composite indicator, Multicriteria analysis, Sub-Saharan Africa

## Abstract

In this article we present datasets used for the construction of a composite indicator, the Social Clean Energy Access (Social CEA) Index, presented in detail in [1]. This article consists of comprehensive social development data related to electricity access, collected from several sources, and processed according to the methodology described in [1]. The new composite index includs 24 indicators capturing the status of the social dimensions related to electricity access for 35 SSA countries. The development of the Social CEA Index was supported by an extensive review of the literature about electricity access and social development which led to the selection of its indicators. The structure was evaluated for its soundness using correlational assessments and principal component analyses. The raw data provided allow stakeholders to focus on specific country indicators and to observe how scores on these indicators contributed to a country overall rank. The Social CEA Index also allows to understand the number of best performing countries (out of a total of 35) for each indicator. This allows different stakeholders to identify which the weakest dimensions are of social development and thus help in addressing priorities for action for funding towards specific electrification projects. The data can be used to assign weights according to stakeholders’ specific requirements. Finally, the dataset can be used for the case of Ghana to monitor the Social CEA Index progress over time through a dimension's breakdown approach.

**Specifications Table**

**Table:** 

Subject	Energy
Specific subject area	Renewable Energy, Sustainability and the Environment
Type of data	Table Figure
How the data were acquired	Queried from Open Data portals, systematically joined and cleaned. Compiled based on a comprehensive horizon-scanning of data sources that are processed for a composite indicator.
Data format	Raw: Formatted data Processed and analysed data
Description of data collection	Raw data was collected by systematic queries. Formatted raw data compilations are utilized for data processing and analyses in the context of the research work.
Data source location	Secondary data in supplementary material. Primary data sources: Moner-Girona, M., Kakoulaki, G., Falchetta, G., Weiss, D.J., and Taylor, N. (2021). Achieving universal electrification of rural healthcare facilities in sub-Saharan Africa with decentralized renewable energy technologies. Joule 5, 2687–2714. https://doi.org/10.1016/j.joule.2021.09.010[2]. USAID (2021). The Demographic and Health Surveys Program STATcompiler. https://dhsprogram.com/publications/index.cfm (accessed July 10, 2021) [3]. WHO (2021). The Global Health Observatory. https://www.who.int/data/gho (accessed July 10, 2021) [4]. FAO (2022). Sustainable Development Goals. Indicators under FAO custodianship database. https://www.fao.org/sustainable-development-goals/indicators (accessed July 10, 2021) [5]. UNDESA (2014). Electricity and education: The benefits, barriers, and recommendations for achieving the electrification of primary and secondary schools. In Energy and Education Journal (Issue 12), pp.1–36 [6]. The World Bank Group (2021). World Bank Indicators. Datasets. https://data.worldbank.org/ (accessed July 10, 2021) [7]. Radboud University (2022). Global Data Lab. GDL Area Database (4.0). https://globaldatalab.org/areadata/ (accessed July 10, 2021) [8]. UNICEF (2021). Monitoring the situation of children and women. https://data.unicef.org/topic/education/covid-19/ (accessed July 10, 2021) [9]. Lardies, C.A., Dryding, D., and Logan, C. (2019). Gains and gaps - Perceptions and experiences of gender in Africa. https://afrobarometer.org/sites/default/files/publications/Policy%20papers/ab_r7_policypaperno61_gains_and_gaps_gender_perceptions_in_africa.pdf[10].
	Rysankova, D., Putti, V. R., Hyseni, B., Kammila, S., & Kappen, J. F. (2014). Clean and improved cooking in sub-Saharan Africa: A landscape report. In Africa Clean Cooking Energy Solutions Initiative. https://documents1.worldbank.org/curated/en/164241468178757464/pdf/98664-REVISED-WP-P146621-PUBLIC-Box393185B.pdf[11]. Siebert, S., Henrich, V., Frenken, K., Burke, J. (2013). Update of the Digital Global Map of Irrigation Areas to Version 5. https://www.fao.org/3/I9261EN/i9261en.pdf[12].
	Moner-Girona, M., Bender, A., Becker, W., Bódis, K., Szabó, S., Kararach, A.G.G., Anadon, L.D.D., Kararach, G., and Diaz Anadon, L. (2021). A multidimensional high-resolution assessment approach to boost decentralised energy investments in Sub-Saharan Africa. Renew. Sustain. Energy Rev. 148, 111,282. https://doi.org/10.1016/j.rser.2021.111282[13]. Bender, A., Moner-Girona, M., Becker, W., Bódis, K., Szabó, S., Kararach, A.G., Anadon, L.D. (2021). Dataset for multidimensional assessment to incentivise decentralised energy investments in Sub-Saharan Africa. Data Br. https://doi.org/10.1016/j.dib.2021.107265[14]. Szabó, S., Pinedo Pascua, I., Puig, D., Moner-Girona, M., Negre, M., Huld, T., Mulugetta, Y., Kougias, I., Szabó, L., and Kammen, D. (2021). Mapping of affordability levels for photovoltaic-based electricity generation in the solar belt of sub-Saharan Africa, East Asia and South Asia. Nat. Sci. reports 11. https://doi.org/10.1038/s41598–021–82638-x[15]. The World Bank (2010). The Global Consumption Database [database]. http://datatopics.worldbank.org/consumption/ (accessed July 10, 2021) [16].
Data accessibility	https://doi.org/10.2905/938F628A-5D2C-408A-B2B9–6E84871665B0 https://data.jrc.ec.europa.eu/collection/id-0076
Related research article	Casati, P., Moner-Girona, M., Shehu, I. K., Szabó, S., and Nhamo, G. (2023). Clean energy access as an enabler for social development: a multidimensional analysis for Sub-Saharan Africa. Energy for Sustainable Development. Volume 72, Pages 114–126 [1].

## Value of the Data

• The data is suitable for constructing a composite index encapsulating multiple indicators related to education, health and wealth that are vital in shaping national and international policies supporting electricity access.
• The raw data is made publicly available and represent a unique resource which allows different stakeholders to identify most appropriate social ecosystem for decentralized electricity access financing or/and funding.
• The data can support stakeholders in monitoring the effects of electricity access programmes on social development by tracking trends over time.
• Stakeholder can tailor the weights assigned to the different dimensions to match their specific requirements. For example, a philanthropic organization may use the Social CEA Index to find regions where funding in electricity generation may have the greatest health benefits. Policy organisation can give weight to factors of specifict policies (poverty alleviation, children well-being, women empowerment etc) to see the specific policy's energy implications.

## 1 Objective

In this article we describe the datasets used for the construction of the Social Clean Energy Access (Social CEA) Index, discussed in detail in P. Casati et al. (2023). The Social CEA is a novel composite index including 24 indicators capturing the status of specific social dimensions related to electricity access for 35 SSA countries. It was created to identify the most suitable countries for funding and implementing decentralised renewable energy systems, shedding light on the opportunities for improving social conditions through clean electrification. In addition, the dataset has been extended to monitor the Social CEA Index trend over time through a dimension's breakdown approach in Ghana. The development of the Social CEA Index was supported by an extensive review of the literature focusing on the relationship between social outcomes and electricity access. A low score of the final indicator implies that financing clean electrification programs is likely to improve specific social outcomes in the identified country. The dataset would allow policy makers, non-for-profit organizations, researchers, and entrepreneurs to potentially re-use the data for tailoring the Index or analyse individual indicators trends, streamlining target countries electrification policies.

## 2 Data Description

This article contains the dataset used for the design and development of the Social CEA Index for Sub-Saharan African countries. The Social CEA Index is built in 5 main dimensions (Healthcare, Education, Gender equality, Quality of life and Economic development), 12 sub-dimensions and 24 indicators. Thus, this dataset focuses on the salient social dimensions related to clean electricity access, further emphasizing the increasingly evident interconnections between energy and the society. The imperative to increase access to clean and affordable energy services is key in promoting poverty alleviation in SSA, thereby contributing to improve social outcomes (e.g. healthcare, education, gender equality), quality of life conditions and economic development.

The description of the datasets is presented in this article, while raw data are provided in the Supplementary Information. The original research article [1] describes the methodology used to create the Social CEA Index, providing evidence about the status of social factors related to electricity access in SSA.

Table 1 summarizes the classification, source, year and description of the 24 indicators composing the Social CEA Index.

**Table 1:** Structure of the social CEA index.

Dimension	Sub-dimension	Indicator name		Data source	Year	Description	Indicator direction
Healthcare	Healthcare facilities	ind.01	Electricity access in health facilities	Moner, M. & Kakoulaki, G. [2]	2021	Percentage of healthcare facilities with electricity access in selected countries. Information on electricity access for healthcare facilities has been collected in the electricity access health facility database (EHFDB). The lower the healthcare facilities with access to electricity the greater the potential for decentralised renewable energies to improve electricity access in these facilities, and thus healthcare outcomes.	Positive
		ind.02	Vaccinated children	USAID [3]	2010–2019	Percentage of children 24–35 months who received all age appropriate vaccinations. The lower the number of vaccinated children, the more beneficial decentralised renewable energy solutions may be in providing electricity to store vaccines.	Positive
	Households healthcare	ind.03	Death caused by HH pollution	WHO [4]	2016	Number of deaths attributable to household air pollution resulting from solid fuels for cooking. Evidence from epidemiological studies have shown that exposure to smoke from incomplete combustion of solid fuels is linked with a range of conditions including acute and chronic respiratory diseases. Of these, evidence for three have been assessed on sufficiently strong basis for inclusion in the burden of disease estimates: acute lower respiratory infections in young children (under 5 years); chronic obstructive pulmonary disease in adults (above 25 years); lung cancer in adults (above 25 years).	Negative
		ind.04	Underweight children	WHO [4]	2010–2019	Prevalence of underweight (weight-for-age <−2 standard deviation from the median of the World Health Organization (WHO) Child Growth Standards) amongst children under 5 years of age. Survey estimates are based on standardized methodology using the WHO Child Growth Standards. Global and regional estimates are based on methodology outlined in UNICEF-WHO-The World Bank: Joint child malnutrition estimates - Levels and trends (UNICEF/WHO/WB 2012).	Negative
		ind.05	Population undernourishment	FAO [5]	2018	Percentage of undernourished people. The higher the incidence of undernourished people the more beneficial decentralised renewable energy solutions may be, in terms of improving both cooking facilities within households and agricultural productivity with positive impacts on nutrition. Thus electricity can be used to make agricultural practices more efficient and refrigerate food produced to store for longer.	Negative
Education	Educational facilities	ind.06	Schools without electricity	UNDESA [6]	2014	Percentage of schools in Africa reporting to have no electricity. The lower the education facilities with access to electricity the greater the potential for decentralised renewable energies to improve electricity access in these facilities and thus educational outcomes.	Negative
		ind.07	Pupil-teacher ratio	World Bank [7]	2010–2019	Average number of pupils per qualified teacher at primary level education in a given academic year. Pupil-teacher ratio is calculated by dividing the number of students at the specified level of education by the number of teachers at the same level of education. Data on education are collected by the UNESCO Institute for Statistics from official responses to its annual education survey.	Negative
		ind.08	Educational attendance	Radboud University [8]	2010–2019	Percentage of children aged 6–8 that currently attends, or in the current school year attended, school. This indicator measures the potential educational impact of bringing electricity to schools; therefore, the impact of binging electricity will be higher where the educational attendance is low.	Positive
	Households education	ind.09	Adults literacy	UNICEF [9], World Bank [7]	2010–2019	Percentage of population aged 15 and older that can both read and write a short, simple statement about their everyday life. This indicator measures the potential educational impact, in terms of literacy, of bringing electricity to communities; therefore, the impact of binging electricity will be higher where the literacy level is low.	Positive
		ind.10	Children with internet at home	UNICEF [9]	2010–2019	Percentage of children in a school attendance age (approximately 3–17 years old depending on the country) that have internet connection at home. Also in this case the indicator relates to the potential educational impact of electrification on children and young people.	Positive
		ind.11	Upper secondary completion rate	UNICEF [9]	2010–2019	Percentage of cohort of young people three to five years older than the intended age for the last grade of upper secondary level of education who have completed that level of education. This indicator measures the potential impact of electricity on youth education, that represents a crucial pillar for the development of a country.	Positive
Gender equality	Women security, health and education	ind.12	Physical/sexual violence on women	USAID [3]	2010–2019	Percentage of women who experienced physical or sexual violence. This indicator focuses on the importance of bringing electricity to public infrastructures, in particular streets. Improving street lighting can make these infrastructures safer especially for women and thus reduce the number of women who experienced any type of violence in public spaces.	Negative
		ind.13	Maternal mortality	UNICEF [9]	2017	Number of maternal deaths during a given time period per 100 000 live births during the same time period. The lower the number of healthcare facilities with access to electricity the greater the potential for decentralised renewable energies to reduce maternal mortality.	Negative
		ind.14	Literate women	USAID [3]	2010–2018	Percentage of women who are literate. This indicator highlights the importance of electricity for women, who spend the majority of their time taking care of the households. Electricity can ease girls and young women from households duties and allow them to attend schools. Thus, the lower the number of households with access to electricity the greater the potential for decentralised renewable energies to improve literacy levels amongst women.	Positive
	Women empowerment	ind.15	Employed women	USAID [3]	2010–2019)	Percentage of women who worked in the 12 months preceding the survey and are working currently. A lower score reflects weaker female emancipation within the labour market, and thus a higher potential impact of electricity access for improving women empowerment.	Positive
		ind.16	Women with internet access	Lardies et al. [10]	2019	Percentage of women with regular access to the internet. The indicator reflects again the importance of electricity for the empowerment of women, allowing them to communicate and share information. Thus, lower indicator scores reflect a potential improvement in women empowerment when bringing electricity.	Positive
Quality of life	Energy access status	ind.17	Electricity access	World Bank [7]	2019	Percentage of population with access to electricity. Data, provided by the World Bank, have been collected amongst different sources: mostly data from nationally representative household surveys (including national censuses) were used. Access to electricity captures the portion of the population who already have access to electricity and therefore indicates a lower potential social impact of electricity access.	Positive
		ind.18	Access to clean cooking	World Bank [7]	2016	Proportion of total population primarily using clean cooking fuels and technologies for cooking. Under WHO guidelines, kerosene is excluded from clean cooking fuels. The indicator measures the potential improvement in the quality of life of population due to electricity and lower levels indicates a greater potential impact of electricity access.	Positive
	Time savings	ind.19	Water accessibility	USAID [3]	2010–2020	Percentage of households with water more than 30 min away round trip. The indicator reflects the crucial role of the water energy nexus and indicates that electricity access could generate greater social impacts when the portion of households living far from water sources is high in a given country.	Negative
		ind.20	Firewood collection time	Rysankova et al. [11]	1998–2012	Average time spent by households on fuel collection daily. The higher the number of daily hours that households spend in collecting fuel, the higher the improvements in the quality of life of communities due to electricity access.	Negative
Economic development	Productive use	ind.21	Area equipped for irrigation	Siebert et al. [12]	2005	Area equipped for irrigation with groundwater, expressed in m^2^ multiplied by GWh/m^2^. Lower values of the indicator indicated that electricity could improve the number of areas equipped for irrigation, and consequently both economic and social outcomes (i.e. healthcare and nutrition).	Positive
	Wealth	ind.22	International Wealth Index	Radboud University [8]	2010–2019	The International Wealth Index is an asset-based wealth index that runs from 0 (no assets) to 100 (all assets) and is comparable across place and time. Lower levels of the Index indicates that electricity access The lower the level of the Index, the greater the potential of electricity access to reduce poverty and foster development.	Positive
	Employment	ind.23	Job creation	Moner-Girona et al. [13], Bender et al., [14]	2019	Estimated number of jobs created directly related to the deployment of PV mini-grids. The indicator was calculated using data on the total MWh of electricity output anticipated if the total number of potential mini-grids were established within each country and the employment factors come from OECD. If the estimated number of jobs created is high, it means that PV mini-grids have a large potential both in terms of deployment and social development.	Negative
	Affordability	ind.24	Affordability of PV electricity	Szabó et al. [15], World Bank [16]	2019	Electricity expenditure per day, in US$. Higher levels of electricity expenditure together with high affordability may indicate that the country has a greater proportion of people with affordable electricity access and thus the social impact of further electrification in those areas will be comparatively more limited.	Positive

Table 2 illustrates the correlation between the “Electricity access” indicator and the remaining 23 indicators.

**Table 2:** Correlation between ind.17 “Electricity access” and the remaining 23 Social CEA indicators.

Table 3 illustrates the Social CEA Index variability under three different stakeholder's perspectives.

**Table 3:** Social CEA Index variability under three different stakeholders perspectives (private sector, international donors and civil society) and according to an equal weights approach.

Table SI.1 shows the description of each indicator and the weights used. Indicators were aggregated according to a weighting system established through a public consultation involving different stakeholders (private sector, public sector and civil society) [17] and the support of internal experts.

Tables SI.2-SI.6 show the methodology used to collect the raw data for the composition of the five dimensions of the Social CEA Index: Healthcare (Table SI.2), Education (Table SI.3), Gender equality (Table SI.4), Quality of life (Table SI.5) and Economic development (Table SI.6).

Table SI.7 contains the original data used as inputs in the COIN tool [18], without data treatment.

Table SI.8 contains the data after winzorization.

Table SI.9 includes the results of COIN tool after calculating the correlations between indicators (Pearson coefficients r) taking into account the direction of effects.

Table SI.10 contains the datasets after the FOREST imputation.

Table SI.11 includes the final dataset related to the trend of the Social CEA Index in Ghana used as inputs in the COIN tool.

Table SI.12 includes the sensitivity analysis, showing the Social CEA Index variability under three different stakeholders perspectives (private sector, international donors and civil society) and according to an equal weights approach.

Table SI.13 contains the Principal Component Analysis (PCA).

**Fig. 1:**
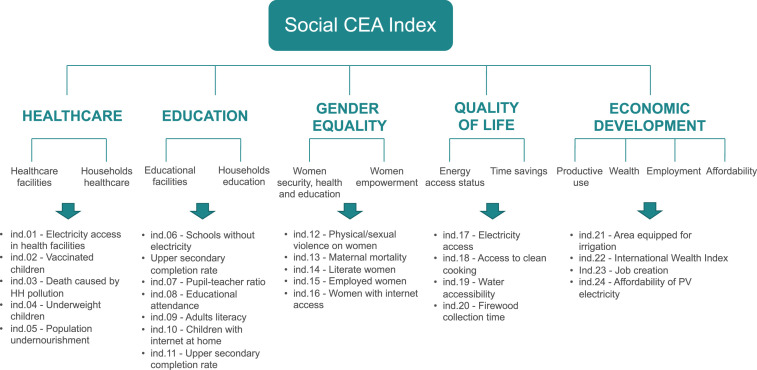
Structure of the social CEA index. Source: Authors’ own elaboration.

Fig. 1 shows the structure of the Social CEA Index.

**Fig. 2:**
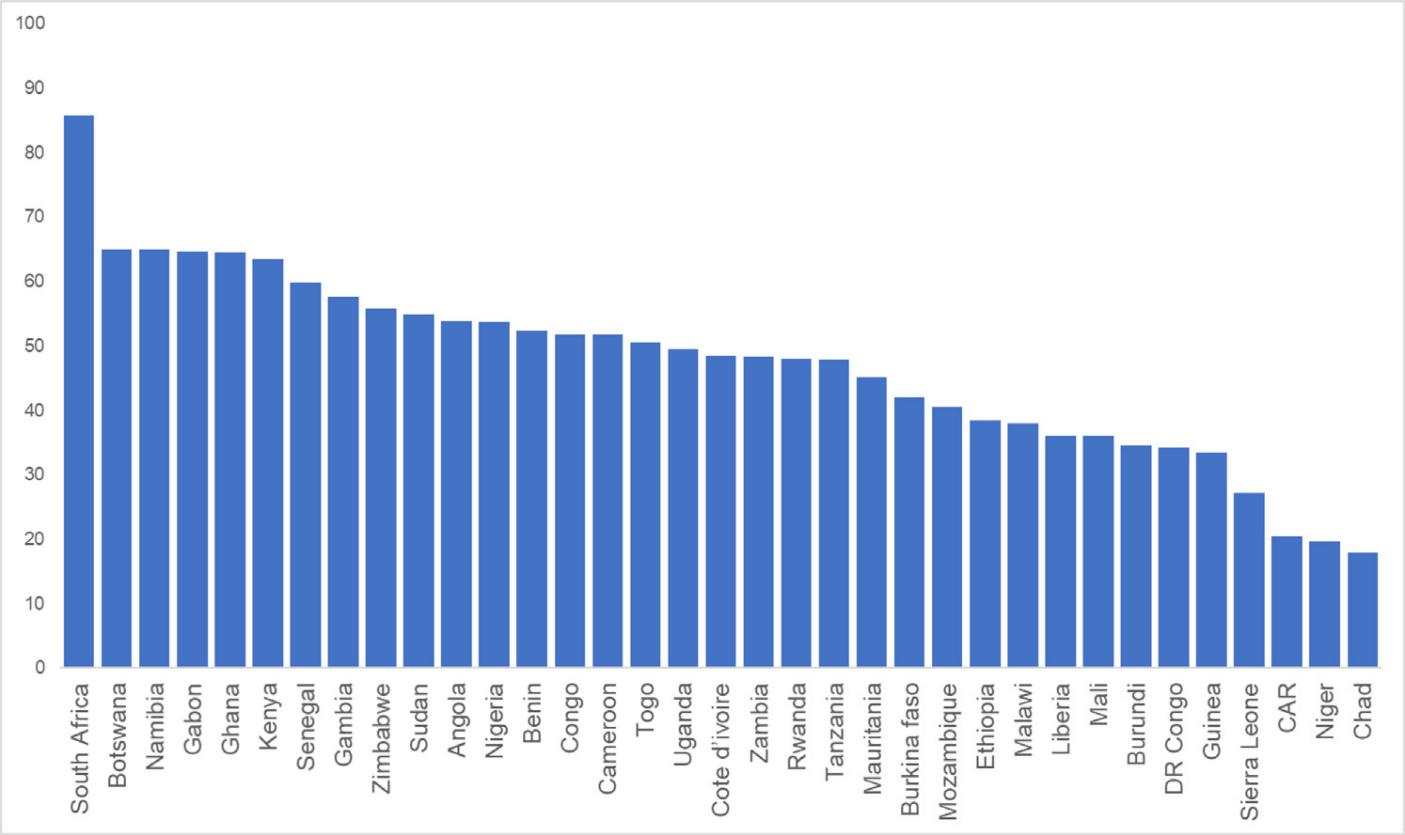
Final Social CEA Index scores. In order to obtain the final Index score data intensification, outlier treatment, missing data imputation, data normalization and indicators weighting and aggregation were carefully carried out.

Fig. 2 shows the Social CEA Index scores.

**Fig. 3:**
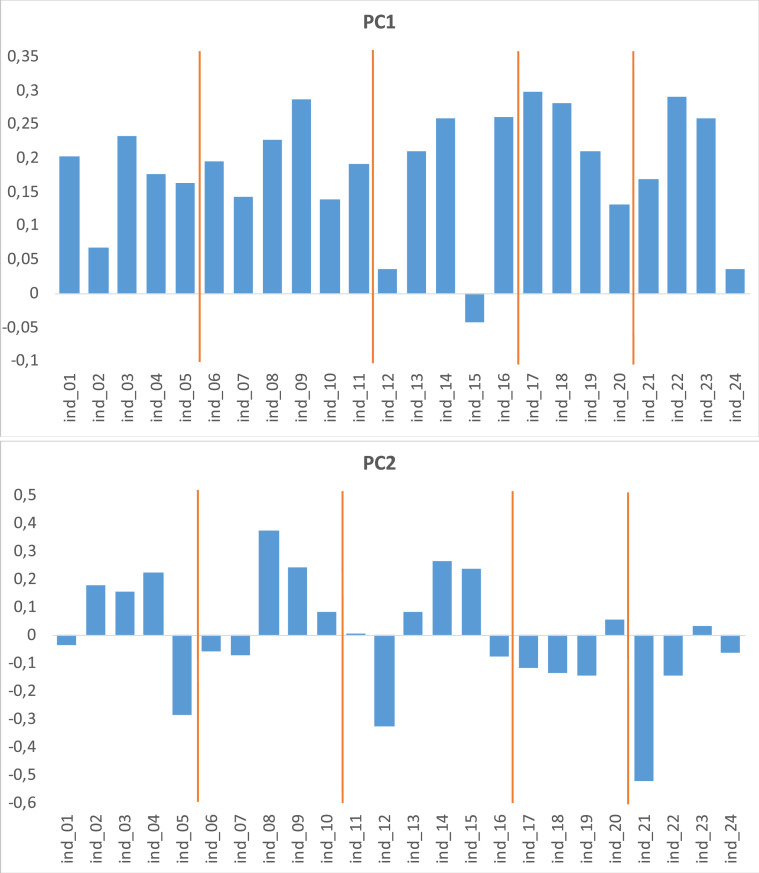
Principal components analysis (PCA). Source: authors’ own elaboration through the software R.

Fig. 3 depicts the Principal Components Analysis (PCA) investigating the underlying structure of the index data, in particular that all indicators contributed to one key measure of social development.

**Fig. 4:**
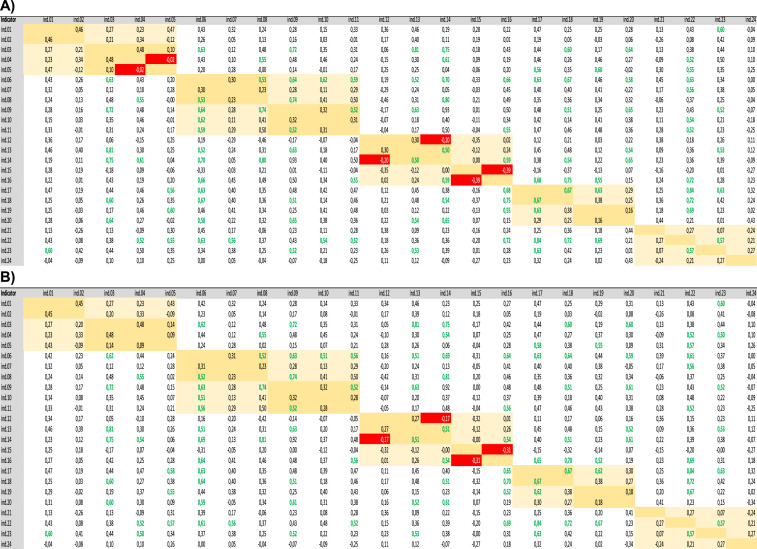
A) Correlational assessments carried out in the COIN tool on the non-imputed data sets. B) Correlational assessments carried out in the COIN tool on the MissForest imputed data sets.

Fig. 4A) displays correlational assessments carried out in the COIN tool on the non-imputed data sets; Fig. 4B) displays correlational assessments carried out in the COIN tool on the MissForest imputed data sets.

**Fig. 5:**
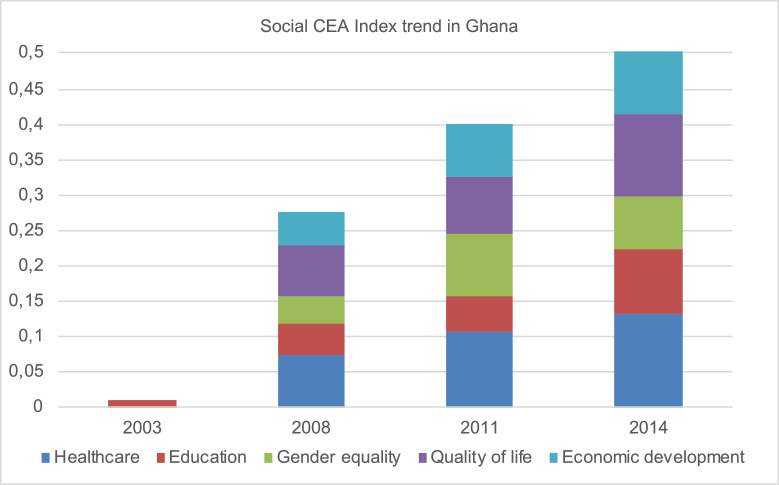
Social CEA index trends in Ghana, also considering dimensions breakdown.

Fig. 5 displays the Social CEA Index scores for Ghana over the selected time frame.

**Fig. 6:**
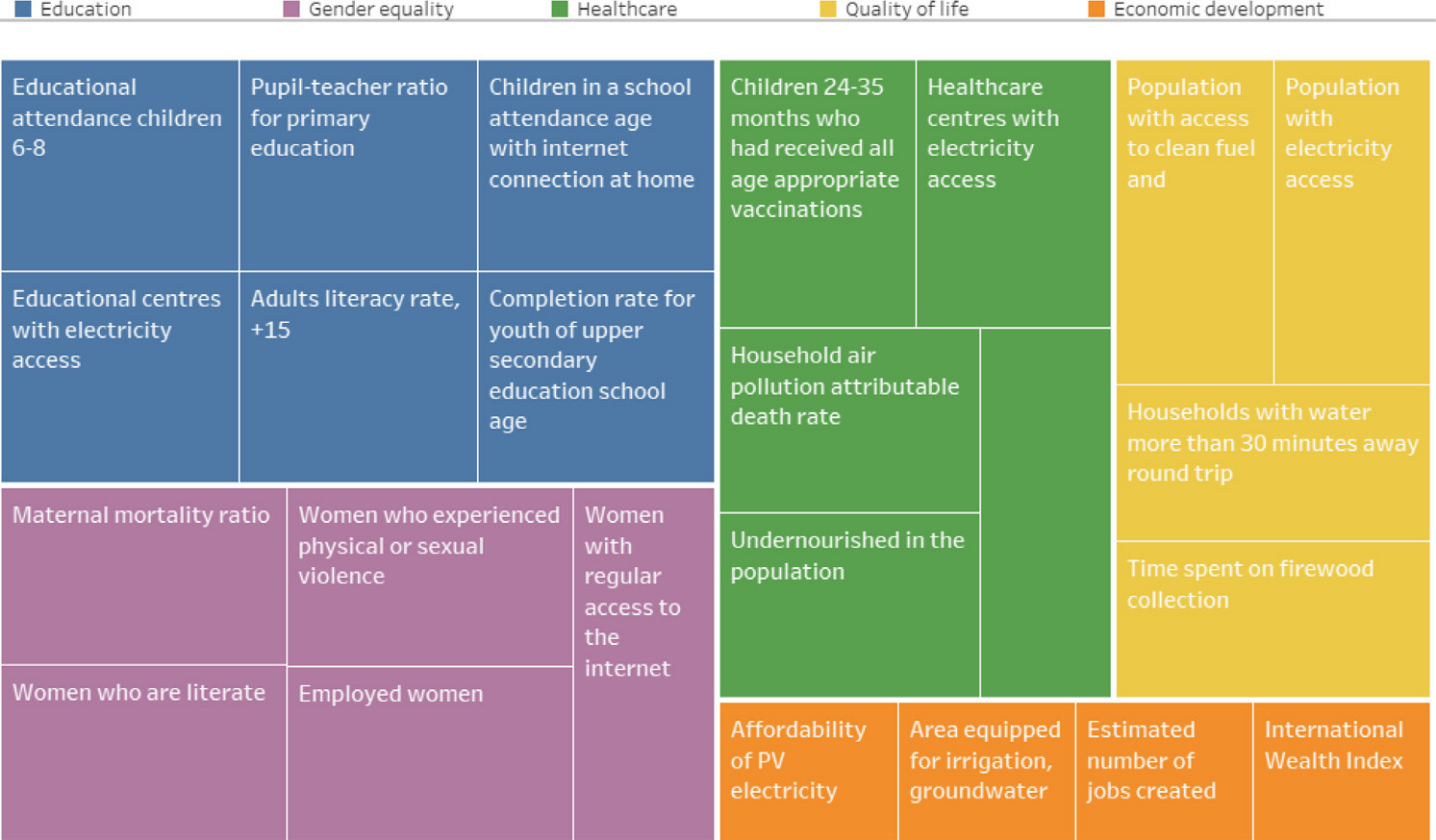
Breakdown of the Social CEA for Ghana.

Fig. 6 represents the breakdown of the Social CEA for Ghana using the attributed weights.

## 3 Experimental Design, Materials and Methods

The Social CEA Index was built in accordance with the “best practice” for composite indicator design outlined by the European Commission's guidance on composite indicators [18]. Its structure was empirically tested and, if possible, improved in terms of accuracy and robustness Fig. 1 [1] illustrates the structure of the Social CEA Index.

The following steps have been completed to ensure raw data were appropriate for use in the final Social CEA Index:

1. The structure of the Social CEA composite indicator was determined prior to data selection. This was done through an extensive review of the existing literature on the social impact of electrification in the context of SSA.
2. Indicator datasets were retrieved from Moner, M. & Kakoulaki, G. (2021) [2], USAID (2021) [3], WHO (2021) [4], FAO (2022) [5], UNDESA (2014) [6], World Bank (2021) [7], Radboud University (2022) [8], UNICEF (2021) [9], Lardies et al. (2019) [10], Rysankova et al. (2014) [11], Siebert et al. (2013) [12], Moner-Girona et al. [13], Bender et al. (2021) [14], Szabó et al. (2021) [15], World Bank (2010) [16] and then grouped according to the identified framework.
3. The datasets were intensified to ensure their comparability across countries. For example, by dividing the indicator by country's population or other metrics.
4. Data processing was then carried according to [18]. To treat outliers, datasets were then winsorized when skew was greater than 2 and kurtosis was greater than 3.5.
5. Countries and indicators with a coverage lower that 63% were removed and then correlational assessments conducted to investigate the underlying structure of the index.
6. Missing data were imputed (i.e. replaced with some substitute value to retain most of the 1information of the dataset) using the MissForest package in the software R and structural assessments were re-run to ensure data-imputation had not significantly altered the underlying structure of the index.
7. In order to bring indicators onto a common scale, rendering them comparable, the dataset was normalised using the min-max method of normalisation.
8. Principal component analysis (PCA) was carried in order to show that all indicators contributed to one key measure of social development.
9. Finally, indicators were aggregated according to the weighting system established through both the results of a public consultation [17] and the support of internal experts. Fig. 2 illustrates the final Social CEA Index scores.

### 3.1 Social clean energy access (Social CEA) index methods

#### 3.1.1 Data selection

Data selection was critical in determining the overall quality of the Social CEA Index. Therefore, to ensure that the datasets used to construct the index were not selected based on convenience, literature review and expert consultations contributed to the development the hierarchical structure of the index prior to data collection. Indicators were chosen from reliable sources and where possible these were collected from International Organisations working under statistical regulations or codes of conduct. The quality of the indicator raw data was assessed using a combination of criteria outlined by the OECD and the European Commission in the “Handbook on Constructing Composite Indicators” [19]. Each of the main dimensions of the indicator was carefully constructed to align with the overall Social CEA composite indicator.

#### 3.1.2 Initial processing

Once the indicator raw data had been compiled, we ensured that indicators were comparable across SSA countries that are characterized with diverse population sizes, land areas, and natural resources. This implied the intensification of appropriate indicators. Data sets were also winsorized, again following the recommendations of the COIN tool for best practice in composite indicator design. This removed the negative impacts of potentially spurious outliers within data sets. Countries missing more then 63% of data across the indicators were removed from the analysis using the COIN tool.

#### 3.1.3 Structural and correlational assessments

To identify the underlying structure of the social composite indicator, both correlational and principal component assessments (PCA) were conducted. Initial correlational investigations were conducted using the COIN tool [18]. These correlational assessments were undertaken to ensure that indicators within the same sub-dimension were not highly correlated (high positive correlation: +0.5), rendering the use of one of them redundant. This was repeated to additionally ensure no indicators were negatively correlated with other indicators in their sub-dimension (high negative correlation: −0.5), which would have suggested an inconsistency between the indicators and what was being measured. Indicators that were either positively or negatively correlated with their neighbors were investigated to determine whether there was a theoretical grounding for this. In the Social CEA Composite Indicator negative correlations were retained only within the gender equality dimension, albeit none of these exceeded −0.5. Furthermore, after the structural assessments, four indicators pertaining to the quality-of-life dimension were categorized in a new dimension, i.e. economic development, addressing in this way the issue of negative correlations.

Particular attention was devoted to the evaluation of the correlation between the ind.17 “Electricity access” and the remaining 23 indicators (Table 2). Correlations have been identified again using the COIN tool [18] but in this case +0.3 represented the threshold for high positive correlation and –0.3 for high negative correlation. This analysis was essential in order to further evaluate synergies between electricity access and social development.

Finally, PCAs (Fig. 3) were conducted using the software R in addition to the correlational assessments, carried out using the COIN tool, to visualize and better understand the underlying structure of the social composite indicator. In particular, the PCA was undertaken to show that all indicators contributed to one key measure of social development, in addition to the qualitative stakeholder suggestions and literature review. This resulted in a refined composite indicator that was valid both qualitatively and quantitatively.

#### 3.1.4 Imputation of missing data

Then challenge of missing data was also addressed. For imputing missing values two different methods can be adopted:

I. Multiple Imputation via Chained Equations, i.e. MICE)
II. Implementation of a random forest algorithm, i.e. MissForest)

Considering the results obtained from [14] and [20] we decided to implement a random forest algorithm (MissForest). In fact, MissForest made fewer assumptions about the shape of each dataset and did not require a specific regression model to be specified for imputation.

Then, structural assessments were re-run to ensure that data imputation had not significantly altered the underlying structure of the index. Fig. 4A and B show the correlational assessments carried out in the COIN tool on the non-imputed data and on the MissForest imputed datasets.

#### 3.1.5 Normalization

The completed data sets were normalised to ensure comparability between indicators originally existing at different scales and ranges, and measured in disparate units. Considering the results provided by [13] and [14], we selected the rescaling or min-max method of normalisation because this preserved the shape of the data distribution for each indicator and did not disproportionately reward or punish exceptional indicator values in contrast to methodologies using Z-scores.

#### 3.1.6 Aggregation and sensitivity assessments

Indicators were aggregated according to the weighting system developed in [1]. We did not opt for an equal weights approach, due to the presence of some social indicators having greater importance in directing financing in decentralised renewable energy systems. Thus, the adopted weighting system was based on the results of a public consultation carried out through a survey [17] and the support of internal experts. Then weights were multiplied by the country's score for each indicator, and then scores across all the 24 weighted indicators were summed together to produce a country's final index score (Fig. 2). A sensitivity analysis was carried out to check whether the scores (and the associated inferences) were robust with changes in stakeholder perspectives (Table 3) [1].

### 3.2 Social CEA Index in Ghana

Finally, a dataset attempting to analyse the Social CEA Index trend was also developed. This was done in order to assess the Social CEA Index trends in a chosen time frame, also according to a dimension breakdown. The lack of complete time series data for several individual indicators limited the possibility of observing the evolution of the Social CEA Index for all countries. Therefore, only the case of Ghana was analysed. Following the methodology adopted for the construction of the Social CEA Index, data have been normalized through the min-max method and the lowest values have been assigned to zero; this explains the low starting scores in 2003 Due to data availability issues, the Index include only 15 out of 24 indicators (Fig. 5).

Fig. 6 illustrates the breakdown of the Social CEA in Ghana. The size of the coloured squares represents the overall weights of the dimension (Healthcare, Education, Gender equality, Quality of life and Economic development) and the size of each square the weights of the individual indicator.

## Ethic statement

The authors declare that the work meets the ethical requirements for publication in Data in Brief and does not involve studies with animals and humans.

## CRediT authorship contribution statement

**Paola Casati:** Conceptualization, Methodology, Data curation, Investigation, Visualization, Writing – review & editing. **Magda Moner-Girona:** Conceptualization, Methodology, Investigation, Visualization, Writing – review & editing. **Ibrahim Khaleel Shehu:** . **Sandor Szabó:** Investigation, Writing – review & editing. **Godwell Nhamo:** Investigation, Writing – review & editing.

## Declaration of Competing Interest

The authors declare that they have no known competing financial interests or personal relationships which have or could be perceived to have influenced the work reported in this article.

Disclaimer: The views expressed are purely those of the authors and may not in any circumstances be regarded as stating an official position of the European Commission.

## Data Availability

Social Clean Energy Access Index (Original data) (Joint Research Centre Data Catalogue).

## Appendix Supplementary materials

Image, application 1
